# Daytime sleepiness among health workers affected by COVID-19 during the “OMICRON” wave

**DOI:** 10.1192/j.eurpsy.2023.2349

**Published:** 2023-07-19

**Authors:** Z. Athimni, M. Mersni, H. Ben Said, G. Bahri, D. Brahim, N. Mechergui, I. Yousssef, S. Ernez, N. Ladhari

**Affiliations:** 1 Department of Occupational Medicine, Charles Nicolle Hospital of Tunis, Tunis, Tunisia

## Abstract

**Introduction:**

Excessive daytime sleepiness is a frequent symptom in the general population. It may be fleeting, due to transient circumstances, or it may be related to certain pathologies. Indeed, following their infection with SARS-COV2, several healthcare workers (HCWs) have complained of excessive daytime sleepiness.

**Objectives:**

This study was conducted to assess excessive daytime sleepiness in the SARS-COV2-affected HCWs during the “OMICRON” wave.

**Methods:**

Cross-sectional descriptive study, conducted among the HCWs of Charles Nicolle Hospital with COVID-19 during the period from 22 December 2021 to 31 January 2022. Sleepiness was assessed using the Epworth Sleepiness Scale administered during the return to work medical visit.

**Results:**

During the “OMICRON” wave, 58 HCWs joined our study. The average age was 39 +/- 10 years. The sex ratio (M/F) was 0.2. The participants had no previous history of sleep disorders. Excessive daytime sleepiness was found in 21 participants (36% of cases). Excessive daytime sleepiness was mild in 81% of cases, moderate in 14%, and severe in 5%. The category most affected was senior technicians in 57% of cases. Most of the HCWs suffering from daytime sleepiness were working in the gynecology department (19%) and the neurology department (19%).

**Conclusions:**

Early and systematic screening for sleep disorders after any SARS-COV2 infection is necessary to ensure the good health of the HCWs and to reduce accidents and errors in professional procedures.

**Disclosure of Interest:**

None Declared

